# Critical Assessment of RNA and DNA Structure Predictions via Artificial Intelligence: The Imitation Game

**DOI:** 10.1021/acs.jcim.5c00245

**Published:** 2025-03-30

**Authors:** Christina Bergonzo, Alexander Grishaev

**Affiliations:** † Biomolecular Measurement Division, Material Measurement Laboratory, National Institute of Standards and Technology, Gaithersburg, Maryland 20899, United States; ‡ Institute for Bioscience and Biotechnology Research, Rockville, Maryland 20850, United States

## Abstract

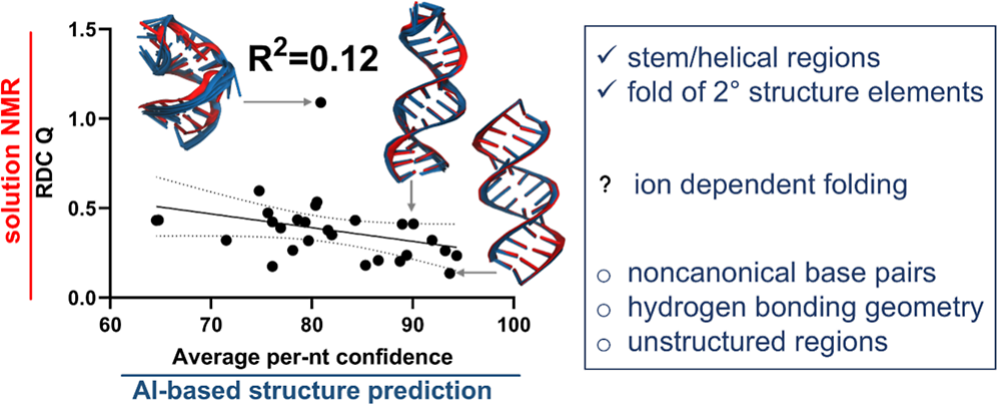

Computational predictions of biomolecular structure via artificial intelligence (AI) based approaches, as exemplified by AlphaFold software, have the potential to model of all life’s biomolecules. We performed oligonucleotide structure prediction and gauged the accuracy of the AI-generated models via their agreement with experimental solution-state observables. We find parts of these models in good agreement with experimental data, and others falling short of the ground truth. The latter include internal or capping loops, noncanonical base pairings, and regions involving conformational flexibility, all essential for RNA folding, interactions, and function. We estimate root-mean-square (r.m.s.) errors in predicted nucleotide bond vector orientations ranging between 7° and 30°, with higher accuracies for simpler architectures of individual canonically paired helical stems. These mixed results highlight the necessity of experimental validation of AI-based oligonucleotide model predictions and their current tendency to mimic the training data set rather than reproduce the underlying reality.

## Introduction

As revealed in the 2018 and 2020 Critical Assessment of Structure Prediction trials,^1^ artificial intelligence (AI) based protein structure prediction outperformed, by a wide margin, the entirety of competing approaches, prevailing over groups of researchers with decades of experience in the field. It suggested feasibility for AI to perform scientific discovery on par with high-resolution experimental structures,^2^ with job executions via open servers taking minutes compared to years for experimental structure determination. A 2024 update, the AI-based program AlphaFold3 (AF3),^3^ pushed these capabilities even further, extending structure predictions to complexes and oligonucleotides, in effect covering the majority of biomolecular research targets. Structures are predicted based on input sequence using transformer neural networks, sequence homology, and by taking advantage of ≈200,000 coordinate sets in the Protein Data Bank (PDB),^4^ accumulated over 5 decades of experimental research.

These developments pose a crucial question: does application of AI for discovery of biomolecular structure generate new knowledge and better insight into reality, or is it producing an elaborate mimic of its training data set? The answer to this question requires a test akin to Alan Turing’s imitation game:^5^ would it be possible to distinguish AI-based predictions from models that relied on experimental data? This question hinges on the notion of accuracy as a measure of a model’s agreement with the underlying reality. As ground truth is rarely known with certainty, one cannot evaluate a distance to an unknown. Therefore, a suitable metric is a measure of a model’s predictive power, i.e. its ability to reproduce external experimental data recorded on the object of study. Such validations have been carried out for proteins,^6,7^ relying on the residual dipolar coupling (RDC) data from solution nuclear magnetic resonance (NMR). In this study we focus on AI-based structure predictions of oligonucleotides, comparing them against experimental NMR observables, and gauging their responses to established effects of changes in buffer’s ionic composition, or primary sequence modifications.

## Materials and Methods

AI-based predictions were made by submitting the RNA/DNA sequences to the Google DeepMind AlphaFold server at https://alphafoldserver.com/ for random seed job executions. Aside from the investigations of the buffer cation impacts, no ion selections were used for model predictions. AlphaFold’s pLDDT scores were extracted from the *B*-factor columns of coordinate files and averaged for all atoms in each nucleotide. The percentage of nucleotides predicted with low confidence was calculated as the ratio of the number of residues with average pLDDT below 70% and the total number of nucleotides in the sequence. AI-generated quintuplets of models were processed via the Reduce software,^8^ to add hydrogen atoms for RDC processing and superimposed by best-fitting to the model displaying the lowest average heavy atom coordinate root-mean-square deviation (r.m.s.d.) to the rest.

NMR structures and RDC restraints were downloaded from the RCSB Protein Data Bank at https://www.rcsb.org/. NMR restraint files were processed to remove all RDCs excluded by the deposition authors from structure determination, retaining only the 1-bond C–H and N–H vectors as those corresponding to highest-precision measurements. The models declared to be the best representative conformers in the ensemble, as listed by the deposition authors in the PDB file header, were used for all further analysis and comparison with the AI-generated results. Coordinates of the most representative NMR conformer were compared to those of the AI-predicted bundle by minimizing the r.m.s.d. between the corresponding non-hydrogen atoms and averaging over the AI-derived bundle. Coordinate dispersion within the AI-predicted bundle was calculated by reference to the mean determined by best-fitting to the model displaying the lowest non-hydrogen atom r.m.s.d to the rest.

RDC data, scaled if necessary for the relative magnitudes of the static dipolar couplings, were fitted to the best NMR conformers and the AF3 bundles via singular value decompositions (SVD).^9^ In the latter case, the interatomic vector orientation (“A”) matrices were averaged over the aligned AI-predicted bundles, with fit results representing the entire ensembles of the output AI-generated modes. RDC data fit quality was reported via both the r.m.s.d. between the experimental and the fitted RDC values, and the *Q*-factor,^10^ defined as
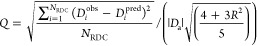
where *D*_a_ is the magnitude of the fitted alignment tensor and *R* is its rhombicity. In the case of 2GBH, RDCs for the loop nucleotides were analyzed by SVD-fitting the alignment tensor to the stem nucleotide RDCs and predicting RDCs for the ensemble of the loop nucleotide conformations with that alignment tensor.

Orientational error optimizations were performed by 100,000 random samplings of normally distributed errors in the experimental RDCs and the nucleotide orientations, by reference to the most representative NMR model. For the RDC data noise sampling, the NMR model-predicted RDCs were generated by the SVD fit of the experimental RDCs and subjected to random additions of normally distributed random error with the standard deviation corresponding to the residual r.m.s. fit of the experimental RDC data. For the samplings of the nucleotide orientations, each nucleotide in the RNA/DNA sequence was subjected to random rotations around its center of mass with respect to randomly chosen axes with normally distributed rotation angles at a given standard deviation. The standard deviations for these random rotations were then optimized to match in the course of 100,000 samplings, either the *Q*-factors or the r.m.s. deviations of the RDC fits of the AI-generated bundles to the experimental RDC data. Overall r.m.s. orientational errors were calculated by averaging the *Q*-factor based and r.m.s based orientational errors.

The widths of the major and minor grooves, the helical axis bending parameters, and the base pair parameters were calculated from the atomic coordinates via the Curves+ and 3DNA.^11,12^ These results are reported in the Supporting Information.

## Results

### Response of AI-Based RNA Predictions to the Introduction of Metal Ions

RNA folding is affected by both monovalent and divalent metal ions, as they associate with the negatively changed backbone phosphates. We gauged the response of AI structure predictions for RNA to the introduction of Na^+^, K^+^, and Mg^2+^ ions for several cases where such effects were established from experiment.

**Figure 1:**
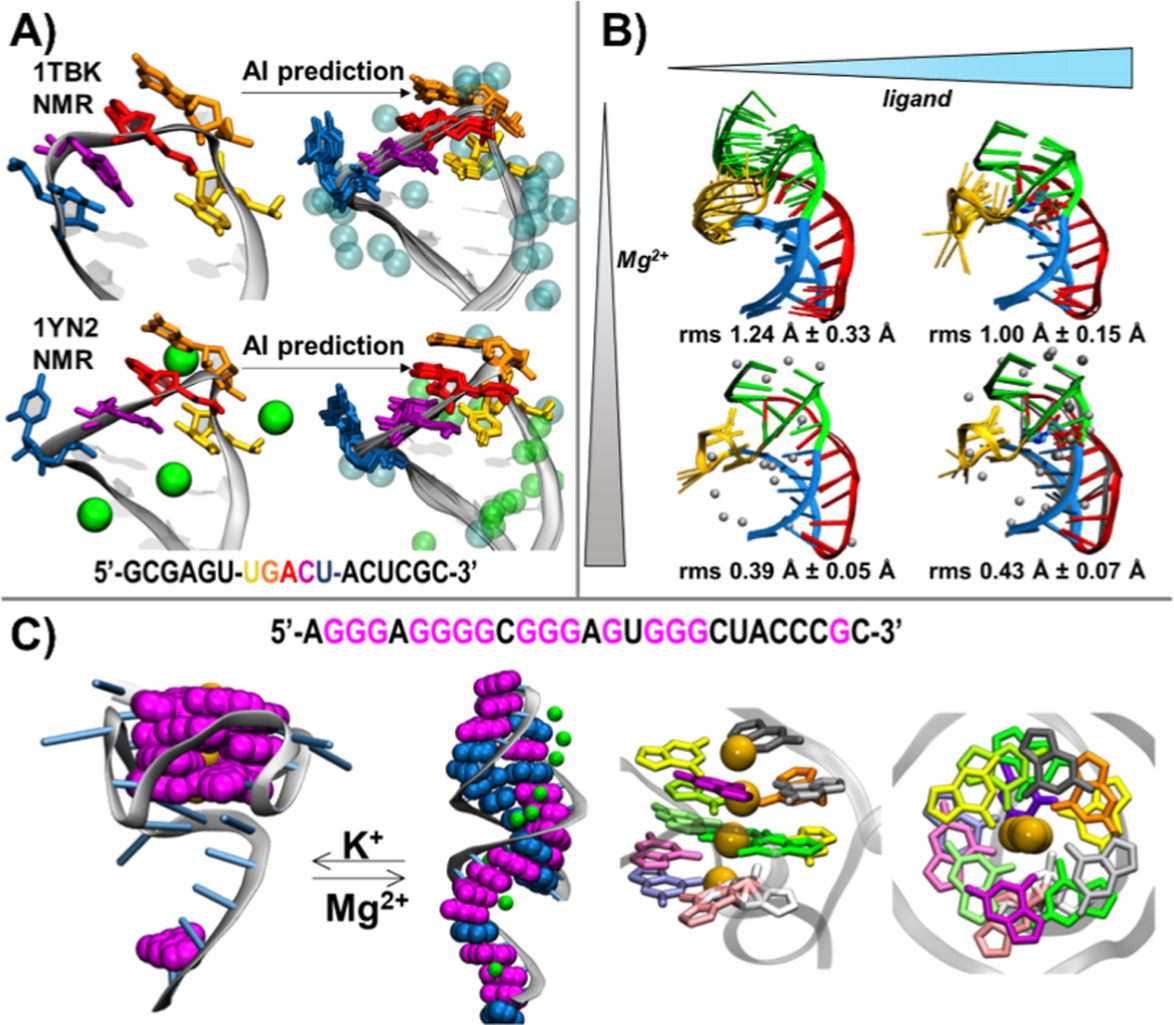
AI predictions of the effects of the cations. Unless labeled as “NMR,” all shown structures are AI predictions. (A) SLV RNA of Varkud satellite ribozyme determined via NMR (left column), without Mg^2+^ ions (top, 1TBK) and with Mn^2+^ ions (bottom, 1YN2), and predicted via AI (right column), with Na^+^ ions and without Mg^2+^ ions (top), and with Mg^2+^ ions (bottom). The UNR motif is colored in yellow, orange, and red, respectively, and the rest of the coloring matches a previous publication (Bergonzo et al. 2015).^15^ Na^+^ ions are colored cyan and Mn^2+^/Mg^2+^ ions are colored green. (B) preQ1 riboswitch in the absence (left column) and presence (right column) of ligand (mimicked using guanosione-5′-diphosphate), and in the absence (top row) and presence (bottom row) of Mg^2+^ ions. preQ1 is colored by domain based on a previous publication (Suddala et al. 2015).^18^ (C) AI-based structure predictions for a designed RNA sequence result in a G-quadruplex in the presence of K^+^ ions, and a hairpin in the presence of Mg^2+^ ions (left panel). Guanine bases are colored magenta, other bases are colored blue, Mg^2+^ ions are colored green, and K^+^ ions are colored orange. Each tetrad is identifiable and the overall helical twist is preserved (right panel, Guanine bases colored by residue number).

Stem-loop V of the Varkud Satellite ribozyme exhibits two conformations depending on the buffer cations (Table 1 and Figure 1a left column): a looser variant of the U-turn (UNR) motif with Na^+^ (PDB ID: 1TBK), lacking the hydrogen bond between R 5′–phosphate and U/H3 and their phosphate/base stacking,^13^ and a canonical compact U-turn observed with Na^+^ and Mn^2+^ as a Mg^2+^ mimic (PDB ID: 1YN2).^14^ AI-based predictions without Mg^2+^ and with Na^+^, resemble the compact U-turn experimentally observed with Mn^2+^, rather than the loose U-motif observed at the experimental conditions with sodium chloride buffer present (Figure 1a top right). With Na^+^ and Mg^2+^ added, AI-generated models lose structure in the stacked bases directly after the turn, as well as stacking between the U base and the R 5′ phosphate (Figure 1a bottom right). AI modeling with only Mg^2+^ added leads to the loss of several of U-turns’ critical characteristics including positioning of the U base and R 5′-phosphate, the hydrogen bond between the U:2′OH and R/N7, and the sharp turn at the N nucleotide (Figure 1 and Table 1 Supporting Information). These deviations are accompanied by inconsistency of the AI-placed Mg^2+^ ions with their locations in 1YN2. No predicted Mg^2+^ ion position matches the experimentally determined sites while Na^+^ ions associate at binding sites between the U and N residues’ phosphate groups and G8/N7, which is a presumed artifact of Mn^2+^ d-orbital interactions^15^ and likely unrepresentative of Mg^2+^ binding. In summary, AI-based predictions without Mg^2+^ recover compact rather than the expected loose U-turn, and the addition of Mg^2+^ in the presence of Na^+^ mostly preserves agreement with the experiment of the RNA conformation, but fails to recover Mg^2+^ binding sites. Mg^2+^-only predictions exhibit further deterioration of conformation accuracy (Supporting Information Figure 1).

**Table 1:** U-Turn Characteristics of SLV RNA^a^

U-turn characteristic	1TBK NMR (+Na^+^/–Mg^2+^)	AI predicted (+Na^+^/–Mg^2+^)	1YN2 NMR (+Na^+^/+Mn^2+^)	AI predicted (+Na^+^/+Mg^2+^)
turn residue N α (degree)	116 ± 6	194 ± 47	184 ± 38	168 ± 2
stacking bases after turn (Å) N, R	3.63 ± 0.19	3.56 ± 0.05	4.22 ± 0.44	3.63 ± 0.03
stacking bases after turn (Å) R, R + 1	3.65 ± 0.30	3.78 ± 0.06	3.80 ± 0.24	3.86 ± 0.07
stacking U base and R 5′-phosphate (Å)	4.16 ± 0.36	4.48 ± 0.27	3.90 ± 0.64	4.66 ± 0.23
H-bond between U 2′OH and R N7 (Å)	3.36 ± 0.29	3.27 ± 0.16	2.55 ± 0.17	2.95 ± 0.07
U N3 and R 3′-phosphate distance (Å)	8.78 ± 0.31	4.18 ± 0.15	5.14 ± 0.68	4.33 ± 0.14

a Averages and standard deviations are calculated for the NMR (1TBK and 1YN2) and the AI-predicted ensembles.

Class I PreQ_1_ riboswitch folds into an H-type pseudoknot with K^+^ (PDB ID: 2L1V)^16^ or Ca^2+^ (PDB ID: 3K1V),^17^ exhibiting a single-molecule Förster energy transfer (smFRET)-characterized conformational change^18^ in the presence of Mg^2+^. AI-based predictions reproduce this experimental trend. Without ligand and Mg^2+^ ions, AI predictions exhibit increased flexibility (Figure 1b, top left), primarily in P2 pseudoknot and L2 loop regions, as indicated by higher average r.m.s.d. and standard deviation of the five-member bundle. With both Mg^2+^ and ligand, the AI-predicted ensemble is less flexible (Figure 1b, bottom right), while ligand-bound and Mg^2+^-free prediction exhibits intermediate flexibility (Figure 1b, top right). The Guanine “ligand” docks into the correct location and conformation, associating with C17, A30, and U6. For the AI-predicted structures, the values of coordinate r.m.s.d. to mean indicate higher deviations in the absence of ions and ligand, and lower deviations in the presence of Mg^2+^ and ligand, qualitatively agreeing with the smFRET results. However, all AI-predicted models are completely folded, while preQ_1_-I aptamer is known to include a prefolded conformational ensemble with flexible 3′ tail, more pronounced without Mg^2+^. Therefore, we conclude that AI-based predictions partially capture the subtle synergy between RNA, ligand, and the ions in the buffer.

A designed G-rich RNA sequence transitions between a hairpin and a G-quadruplex depending upon the ionic environment.^19^ AI-based predictions for this sequence with added K^+^ or Mg^2+^ ions generally follow that trend (Figure 1c left). The presence of Mg^2+^ promotes a hairpin correctly exhibiting 5 out of 7 base pairs excluding the A–A mismatch and the loop-closing G-C. The 5′ tail is predicted to be helical, which is not observed experimentally. Structure prediction with K^+^ produces a G-quadruplex (Figure 1c right), with K^+^ ions centrally located relative to the three planar tetrads, which exhibit a helical twist. The geometry of each tetrad is maintained by some, but never all of the eight expected hydrogen bonds (Supporting Information Figure 2). In summary, AI-based predictions correctly capture the overall transition while missing a number of important base pairing characteristics.

### Response of AI-Based RNA Predictions to the Primary Sequence Changes

RNA folds exhibit exquisite sensitivity to modifications of the primary sequence, with several examples of small changes at termini producing dramatic rearrangement of the overall structure. We have investigated AI-based predictions for two such cases.

**Figure 2:**
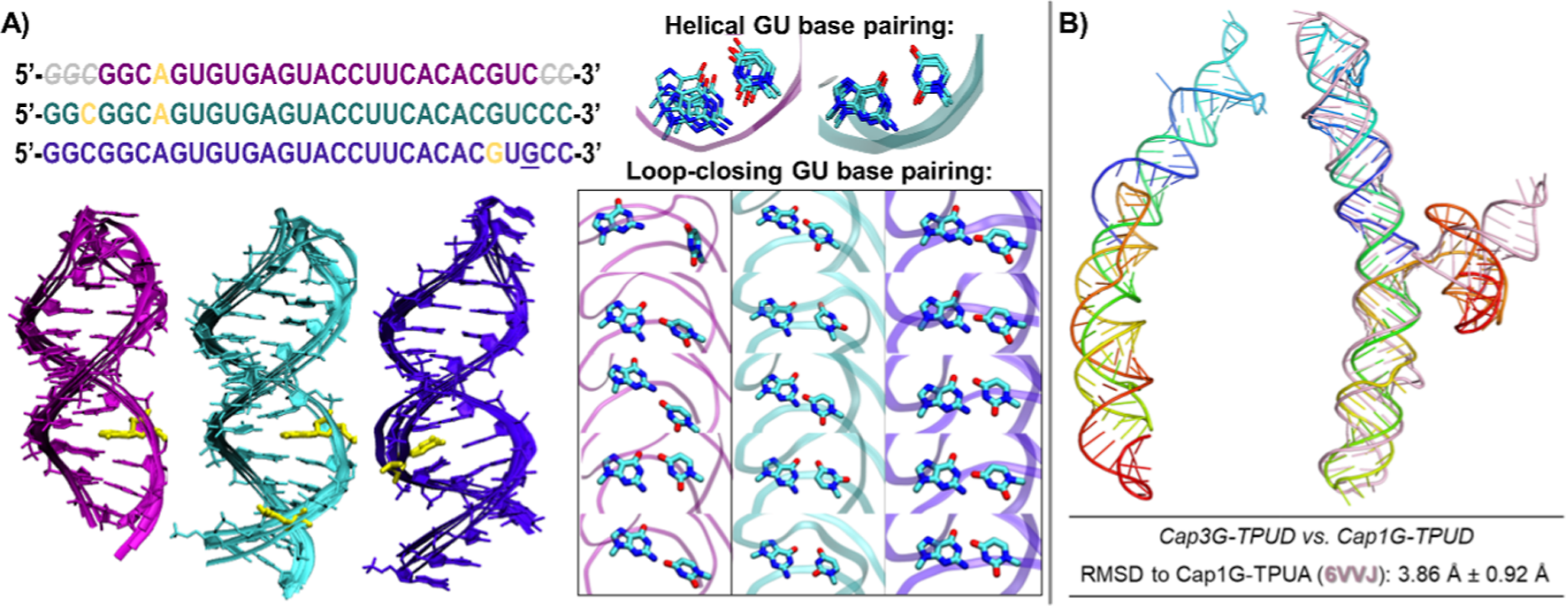
AI-based predictions for the differences in terminal sequences. (A) MAPT 10 exon RNA adopts experimentally established secondary structures (left), including reorganization of the hairpin upon increase in the length from 25 nt (magenta) to 30 nt cyan), and +19G mutation (indigo, 19G underlined in sequence), as well as correct placement of the single-nt bulges (yellow). While the helical GU base pair is canonically formed in the 25 nt and the 30 nt hairpins (A, top right), AI predictions for the loop-closing GU base pair vary (A, bottom right). (B) Comparison of the AI predictions for Cap3G-TPUD (left) and Cap1G-TPUD (right), 142 nt and 140 nt constructs employed to facilitate the NMR resonance assignment for Cap1G-TPUA (PDB ID: 6VVJ). Overlap of the most representative NMR model for Cap1G-TPUA (pink), with the Cap1G-TPUD AI predictions shows good alignment for stacked HIV-1 TAR and poly A domains.

A 25 nt MAPT 10 exon regulatory hairpin folds as a lower stem and an upper stem-loop, separated by an A-bulge. It undergoes lower stem rearrangements upon addition of 3 bases at the 5′-end and 2 bases at the 3′-end, transitioning to a 30 nt hairpin, as demonstrated by single-molecule unfolding with optical tweezers.^20^ A subsequent C-to-G mutation at the +19 position shifts the stem into a 27 nt hairpin. AI-generated models largely agree with the experimentally established secondary structures, which were predicted from primary sequences and validated by single-molecule mechanical unfolding using optical tweezers (Figure 2a left), including hairpin lengths and single-nucleotide bulge locations. The UACC tetraloop capping the hairpin fits no known motif,^21^ and exhibits inconsistent structure predictions for each sequence (25nt, 30nt, +19G-30nt). AI-based modeling of G-U base pairs is uneven, with those embedded in the helical stems for the 25-mer and 30-mer sequences well-formed, and tetraloop-closing G-U pairs either unformed or inconsistent with hydrogen bonding (Figure 2a right). Overall, the fidelity of the AI-generated models is mixed - the secondary structure hairpins are reproduced well while accuracy is lower in noncanonical regions.

HIV transcriptional control element RNA was shown to exhibit dramatic differences in structural organization depending on the nucleotide sequence capping its 5′ terminus.^22^ A single-nucleotide 5′ G overhang allows helix dimerization of TAR with the polyA region, while a 5′ 3G cap inhibits such dimerization, and promotes a TAR stem loop with an unstructured polyA region. AI-based modeling predicts an extended stacked helical structure for both 3G and 1G caps (Figure 2b). In predictions of the 3G cap, the polyA region is always incorrectly structured and the correct dimer interface is never formed. However, the Cap1G-TPUD generally agrees with an analogous stacked TAR-polyA structure (Cap1G-TPUA, PDB ID: 6VVJ).

### Validation of AI-Based RNA and DNA Structure Predictions via Experimental RDCs

AI-based structure predictions were carried out for 28 RNA and DNA constructs previously studied via solution NMR at weakly aligned conditions, with structure and experimental data depositions in the PDB.^23−42^ As 75% of RNAs in our set correspond to individual hairpins or stems, they are biased toward RNA’s most basic building blocks, well-represented in the PDB and likely constituting best-case scenarios for structural accuracy. With NMR structures of RNA impacted by the restraint density and the refinement force-field,^43^ we investigate AI-based models via their agreement with experimental RDCs, connecting them to geometric measures of accuracy via corresponding orientational errors. This metric represents r.m.s. deviation in the orientations of individual nucleotides that would be required to match the observed agreement with the experimental RDCs relative to a target structure. Compared to estimates based on random interatomic vectors,^44^ our procedure employs both the structure and the atomic identities of the measured RDCs, accounting for nonuniform vector distributions and interatomic vector correlations in individual nucleotides.

**Figure 3:**
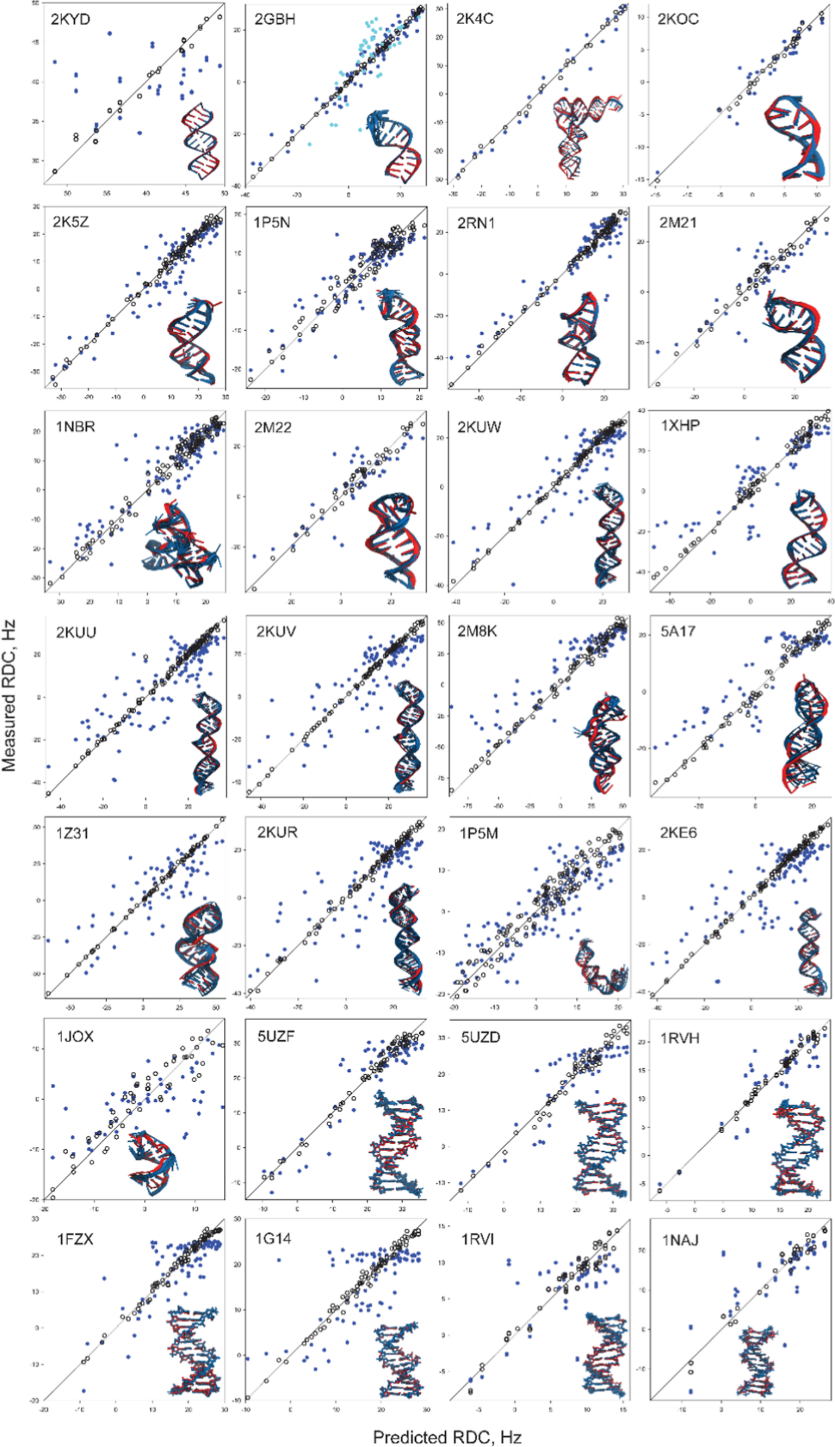
RDC-based validation of the AI predictions for 28 tested RNA and DNA constructs. PDB IDs are as listed. Most representative NMR models (red) are shown aligned with the AI-generated bundles (blue). NMR structure-predicted RDCs are depicted as open black circles and AI model-predicted RDCs as blue filled circles. For 2GBH, predicted RDCs are shown in blue for the stem and in cyan for the loop.

The results of these RDC-based validations are summarized in Figure 3, Table 2, and Supporting Information Table 2, exhibiting r.m.s. orientational errors ranging from 7° to 30°. The highest accuracies, with sub-10° errors are observed for the simplest, completely and canonically base-paired helical stems including 2KYD and 2GBH-stem for RNA, and 5UZD and 5UZF for DNA. Four of our test cases exhibit r.m.s. orientational errors exceeding 20° (1JOX, 2KE6, 1P5M, and 2GBH-loop), including two stem-loops, a helical stem connected to a stem-loop via an internal loop, and a dynamic eight-nucleotide loop capping a stem. Overall, our test set yields AI models’ r.m.s. orientational errors of 15° ± 5°, uncorrelated with the AF3 confidence metrics, but positively correlated with both translational errors and model precision (Supporting Information Figure 3). Some of the test cases are discussed below.

**Table 2:** Parameters of the NMR Models and the AI-Based Predictions for RNA and DNA Constructs Used for RDC-Based Validation

RNA									
PDB ID	AI, pLDDT < 70%	NMR, *R*_gyr_ (Å)	AI, coordinate r.m.s.d. to mean (Å)	AI, coordinate r.m.s.d. to NMR (Å)	NMR, RDC Q	AI, RDC Q	NMR, RDC r.m.s.d. (Hz)	AI, RDC r.m.s.d. (Hz)	AI, orientation error (deg)
2kyd	0.0	15.1	0.17	0.48	0.016	0.135	0.86	6.11	6.6
2gbh stem	0.0	10.4	0.38	0.78	0.037	0.174	0.71	3.44	8.0
2k4c	0.0	24.4	0.16	3.43	0.072	0.234	1.15	3.65	10.1
2koc	0.0	10.4	0.51	0.93	0.078	0.237	0.60	1.66	10.1
2k5z	0.0	15.1	0.89	1.92	0.061	0.264	1.39	5.21	10.7
1p5n	11.8	16.7	0.91	1.82	0.180	0.350	2.38	4.17	13.0
2rn1	0.0	14.7	0.53	1.16	0.068	0.320	1.75	7.12	13.3
2m21	28.6	12.9	1.22	2.38	0.098	0.410	2.34	6.92	13.5
1nbr	3.4	14.5	1.09	2.24	0.122	0.318	2.28	6.20	14.4
2m22	17.4	13.1	0.64	2.29	0.116	0.389	2.41	6.38	14.6
2kuw	0.0	22.0	1.15	1.83	0.044	0.377	1.07	7.58	15.2
1xhp	0.0	16.3	0.53	1.22	0.083	0.412	2.49	10.85	16.3
2kuu	0.0	23.2	1.10	2.08	0.071	0.422	2.02	9.74	16.6
2kuv	0.0	24.3	1.38	1.98	0.029	0.435	0.81	9.95	17.7
2m8k	54.2	17.2	1.03	2.43	0.095	0.433	3.96	16.59	18.2
5a17	56.3	20.5	1.34	4.04	0.114	0.432	2.15	7.68	18.5
1z31	0.0	15.0	1.27	2.57	0.019	0.534	0.82	16.49	18.8
2kur	0.0	23.5	1.81	3.04	0.049	0.473	1.22	9.55	19.1
1p5m	14.5	21.6	1.60	3.16	0.217	0.597	2.55	5.57	20.2
2ke6	0.0	23.6	1.22	2.29	0.042	0.515	1.01	9.62	20.2
2gbh loop	12.5	13.4	1.71	N/A	N/A	0.423	N/A	8.37	22.4
1jox	0.0	10.7	1.32	3.57	0.201	1.091	2.92	6.89	29.5

DNA									
PDB ID	AI, pLDDT < 70%	NMR, *R*_gyr_ (Å)	AI, coordinate r.m.s.d. to mean (Å)	AI, coordinate r.m.s.d. to NMR (Å)	NMR, RDC Q	AI, RDC Q	NMR, RDC r.m.s.d. (Hz)	AI, RDC r.m.s.d. (Hz)	AI, orientation error (deg)
5uzf	0.0	13.8	0.34	0.65	0.052	0.180	1.49	4.71	9.5
5uzd	0.0	13.6	0.33	0.54	0.063	0.208	1.74	4.90	9.8
1rvh	0.0	11.9	0.24	0.84	0.038	0.203	0.67	3.27	11.6
1fzx	0.0	12.8	0.27	0.61	0.043	0.262	1.03	5.44	13.2
1g14	0.0	12.9	0.24	0.54	0.039	0.320	0.94	6.35	15.2
1rvi	0.0	13.9	0.40	1.16	0.097	0.432	1.15	4.03	18.1
1naj	0.0	13.2	0.85	1.33	0.081	0.351	1.67	6.56	18.5

MLV dimer initiation site helical stem (PDB ID: 2KYD)^33^ yields the best validation statistics of all tested AI models, with r.m.s. orientational error of 7° and similar groove width profiles for the NMR and AI models (Supporting Information Figure 4). The apparent high scatter in the RDC correlation plot for 2KYD in Figure 3 reflects the fact that the experimental RDCs sample only approximately 12% of the theoretically accessible range as they are limited to C–H nucleobase vectors, with little angular variation relative to the *z* axis of the axially symmetric alignment tensor. Helix 35 of 23S E. coli rRNA includes a stem (PDB ID: 2GBH) topped by a conformationally disordered octaloop.^28^ The accuracy of corresponding AI-generated models was assessed separately for the rigid Watson–Crick paired stem and the previously uncharacterized flexible loop, yielding respective r.m.s. orientational errors of 8°, second-lowest in the set, and 22°, second-highest in the set.

Iron-responsive element RNA (PDB ID: 1NBR)^24^ contains a lower stem rigidly positioned relative to an upper stem-loop, with a C7 bulge and dynamic nucleotides 15 to 18 within the 13 to 18 hexaloop. Base positioning for the 13 to 17 stretch are qualitatively similar for the NMR and AI models. However, AI-generated models do not reproduce the dynamic nature of nucleotides 15 to 18, with inclusion of those RDCs in validation increasing the fit *Q*-factor from 0.318 to 0.453 (Supporting Information Figure 5). In contrast to the NMR data, AI-based models also exhibit increased variability in the upper stem-loop and flexible positioning between the upper and the lower helices. AI models’ r.m.s. orientational error of 14° is close to the test set average.

Helix II of the template boundary element of Tetrahymena telomerase RNA (PDB ID: 2M22)^36^ comprises a helical stem with a noncanonical A6-A18 pair, capped by a GUAAU pentaloop. Even though AI models include A6-A18 pairing, both the amino hydrogens and the N1 atoms are in close proximity, inconsistent with principles of hydrogen bonding. AI models exhibit the pentaloop stacked with the stem, with G10 and U11 in the major groove, and A13, A14, and U15 in the minor groove. For the NMR structures, while G10 and U11 are in the major groove, none of the bases are stacked and A13 is in the major groove. Fitting the alignment tensor to stem RDCs (*Q*_fit,stem_ = 0.271) and validating the loop RDCs (*Q*_val,loop_ = 0.671), confirms inconsistency of the AI models’ pentaloop with the NMR data (Supporting Information Figure 6). AI predictions’ overall r.m.s. orientational error of 15° is close to the test set average.

Cytoplasmic mRNA transport element (PDB IDs: 2KE6, 2KUR, 2KUU, 2KUV, 2KUW) is a three-segment helix separated by two bulges and capped by an octaloop.^32^ These constructs include the A′-RNA wild-type sequence and four mutants designed for conversion between the A′- and A-RNA conformations. AI models’ orientational errors range between 15° and 20°, with lower accuracies for the A′-conformations of 2KE6 and 2KUR and the highest accuracy for the 2KUW variant with A-RNA lower stem. Major groove width profiles also exhibit differences between AI and NMR models (Supporting Information Figure 7).

Enzyme-activating fragment of human telomerase (PDB ID: 1Z31) contains a lower helix with a bulge, connected via an internal 5-nucleotide loop to a UUCG stem-loop.^27^ The stems and the UUCG loops are consistent for the NMR and AI models, but the internal loop exhibits differences, leading to changes in relative helix positioning and high 18° orientational error.

*Kluyveromyces lactis* telomerase RNA (PDB ID: 2M8K) folds into an H-type pseudoknot including G-C:C and U-A:U triples.^34^ While AF3 models generally reproduce the Watson–Crick paired part of the triplets, positioning of the Hoogsteen-paired purines in the stack is distorted. In result, the AI models exhibit elevated r.m.s. orientational error of 18°.

The SOLE element of Oscar mRNA (PDB ID: 5A17) includes a helical stem with A24 bulge, capped by an AUCAA pentaloop.^38^ NMR data for A24 indicate significant conformational variability, in contrast to its helical stacking for the AI models. Within the pentaloop, AI models exhibit fully stacked bases, with A14 and U15 in the major groove, and C16, A17, and A18 in the minor groove. In contrast, NMR data indicate the absence of well-defined pentaloop structure aside from A14. The 19° r.m.s. orientational error for the AI models is higher than the test set average.

For the P5.1 hairpin of Bacillus RNase P, 1JOX structure reports a novel UGAGAU hexaloop capping a helical stem with stacked-in U14 bulge.^23^ AI models exhibit flipped out bases for U14 and G10, with Watson–Crick edges of G10 and U9 lined up. In contrast, in 1JOX the Hoogstein edge of G10 is lined up with the Watson–Crick edge of U9. The base of A11 is in the major groove in 1JOX and in the minor groove in the AI models. The structure of the hexaloop region in the AI models, distinctly different from 1JOX, resembles a GAGA tetraloop. The overall orientational error of ≈30° is the highest of all cases in our test set.

Even though DNA structures do not exhibit the diversity of RNA’s molecular folds, our tests of AI-generated DNA models yield similar orientational errors of 10° to 19°. The best validation statistics are observed for 5UZD and 5UZF, employed to investigate A-tract groove widths in DNA.^42^ These trends are consistent between the NMR and AI models (Supporting Information Figure 8), with helical axes bent toward the minor grooves.

A pair of self-complementary DNAs containing central AAAATTTT (A_4_T_4_, PDB ID: 1RVH) and TTTTAAAA (T_4_A_4_, PDB ID: 1RVI) sequences^41^ proved to be more challenging for AI modeling. Both NMR-determined and AI-generated helices exhibit bending toward the minor grooves, and narrowing for A_4_T_4_, or widening for T_4_A_4_, of the minor groove toward the middle of the sequences. Minor groove 5′–3′ narrowing is a known feature of DNA A-tracts, well-represented in the PDB. The opposing A-tract orientations in the A_4_T_4_ and T_4_A_4_ sequences result in base stacking differences at the ApT and TpA steps in the NMR models, with high negative roll of −12° and bending toward the minor groove for ApT, and high positive roll of 13° and bending toward the major groove for the TpA. These effects are entirely missing for the AI-generated models. NMR data also indicate bending between the A/T blocks and the flanking G-C base pairs, toward the major groove and via roll for the A_4_T_4_ sequence, and toward the minor groove and via a roll/tilt for the T_4_A_4_ sequence (Supporting Information Figure 9). These bends are present in the A_4_T_4_ AI models, albeit scaled down by 1/2, but missing in the T_4_A_4_ AI models, resulting in differences in helical bending and orientational error of 18° for the AI-predicted T_4_A_4_ construct.

Among our DNA test cases, the highest orientational error of 19° was found for the self-complementary Drew-Dickerson dodecamer (PDB ID: 1NAJ).^39^ With over 40 crystal structures reported in the PDB, it arguably represents the best-studied structure of a DNA. Nonetheless, while the NMR and AI base-pair tilt profiles are similar, roll and groove width profiles are markedly different (Supporting Information Figure 10), possibly reflecting previously noted crystal structure distortions for short oligonucleotides due to lattice and bound cation effects.^39^

**Figure 4:**
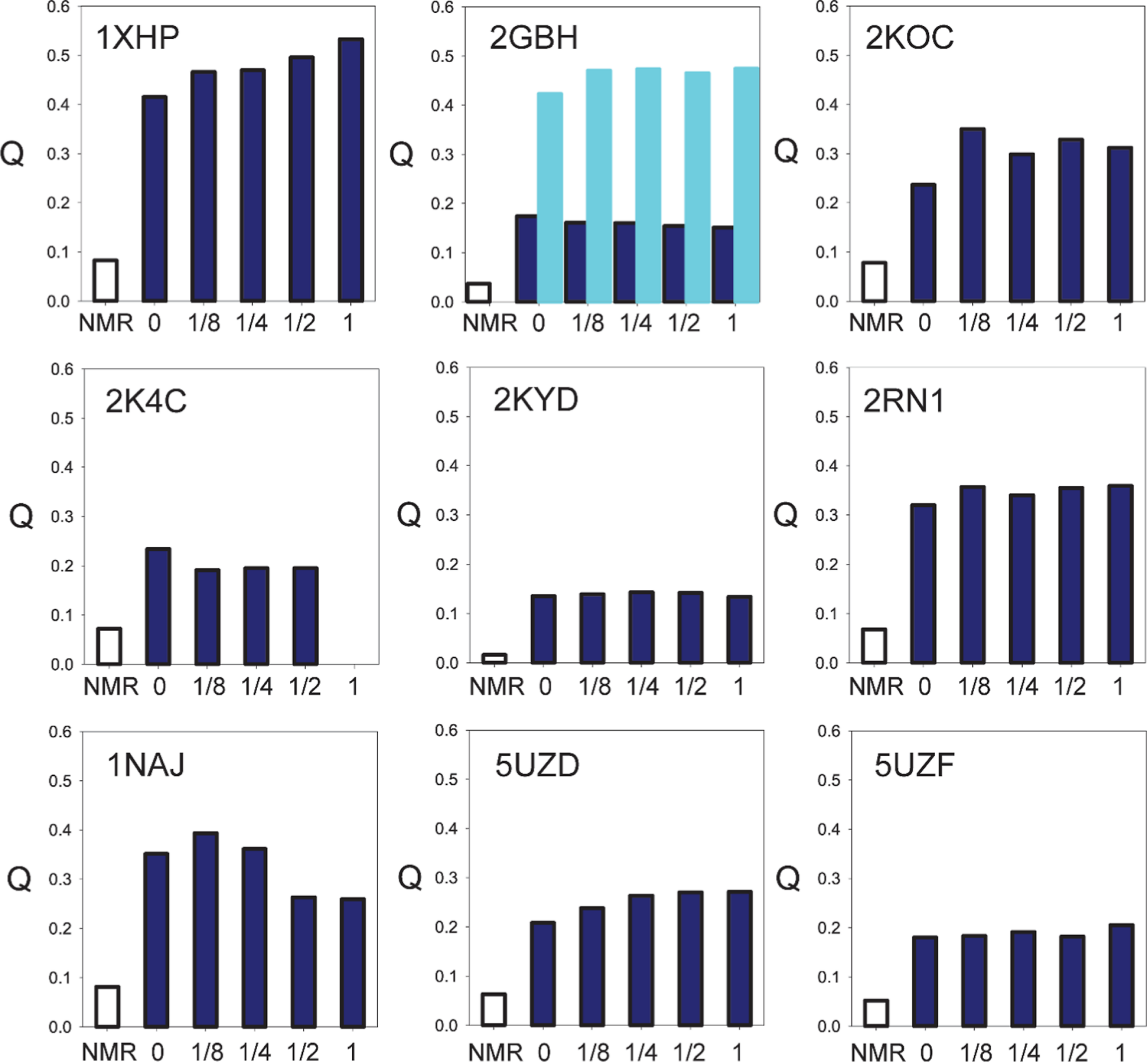
Effect of Na^+^ ion additions on the accuracy of the AI-based predictions for a set of 9 RNA and DNA constructs. PDB IDs are as listed. *Q*-factors for the reference NMR models are shown as open bars and those for the AI-generated model bundles as filled blue bars. For 2GBH, blue bars correspond to the stem and cyan bars to the loop. The ratios between the number of the added Na^+^ ions and the number of nucleotides in the sequences are listed on the horizontal axes. For 2K4C, the 1 Na^+^/nt calculation could not be performed due to limitations on the total number of added ions.

RDC validation was also used to gauge the response of the AI-generated structures of oligonucleotides to the introduction of monovalent cations, present at both physiological conditions and in NMR buffers. Six RNAs and three DNAs from our set were used, with Na^+^ ions added at 1/8, 1/4, 1/2 and 1 ion per nucleotide. As indicated by the RDC fit statistics (Figure 4), we do not observe systematic improvements in the accuracy of the AI-generated structures with the inclusion of Na^+^.

## Discussion

**Figure 5:**
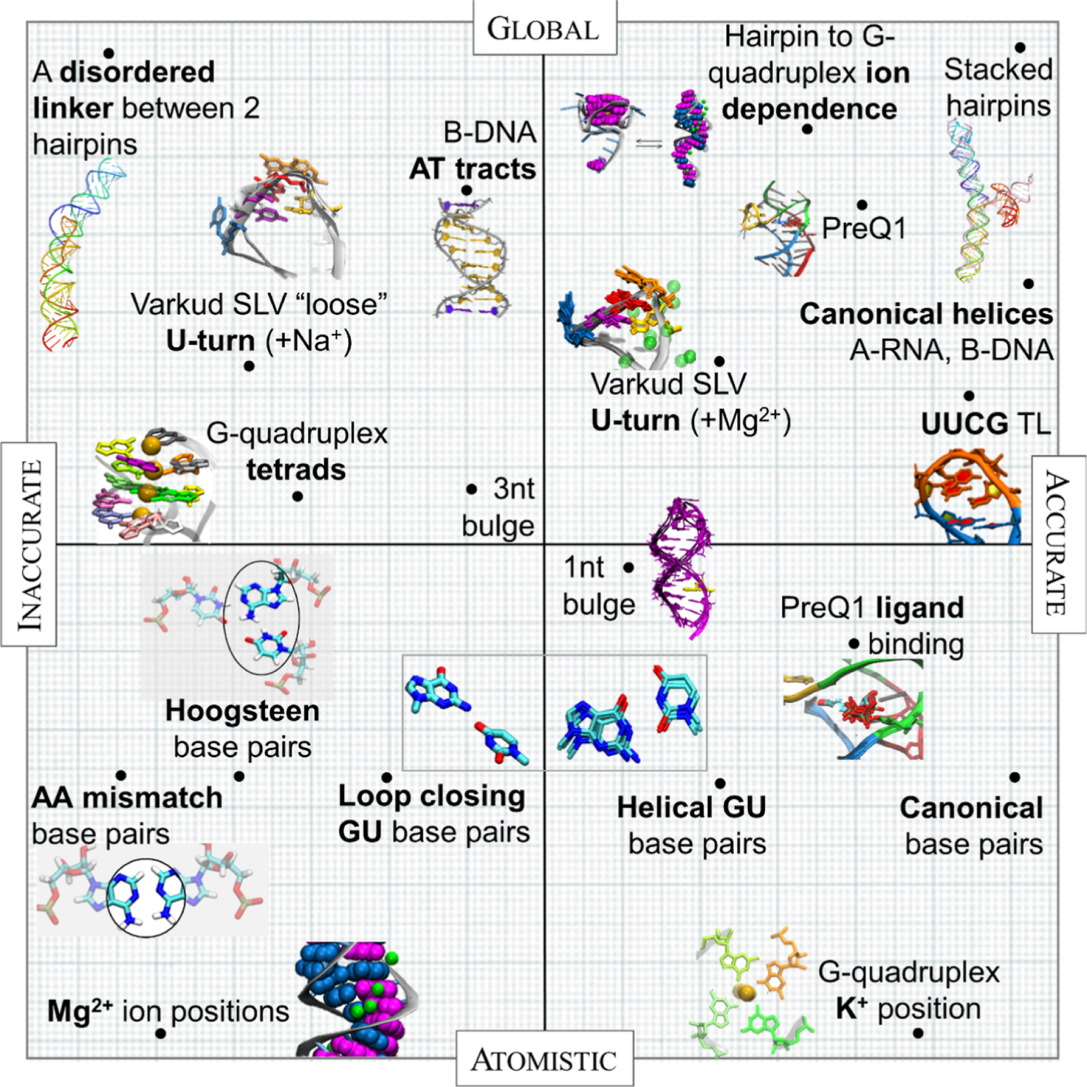
A visual summary of results presented in this work. The vertical axis ranges from global structure to atomistic detail. The horizontal axis ranges from inaccurate to accurate, as determined by comparison to expected conformations or fits to RDCs.

Our assessment of the accuracy for AI-derived models of oligonucleotides reveals a hit-or-miss performance, with some structural aspects reproduced well, such as canonical base pairing, common short RNA loops, or DNA A-tracts, while less common or longer loops, dynamics, or details of hydrogen bonding are not as consistent with reality (Figure 5). We observe a lack of consistent correct response to introduction of cations or sequence modifications known to affect structure. When assessed against RDCs, highest fidelity is observed for more basic structural elements, with accuracy deteriorating with the architectural complexity. AI-based model predictions for individual oligonucleotide helices exhibit 7° to 10° r.m.s. orientational errors. In the presence of multiple conformations, or incorrect loop predictions, orientational errors reach 20° to 30°. Deterioration of AI modeling accuracy for RNA loops is expected to lead to errors in predictions for multihelix sequences, or binding partner interactions. Orientational errors determined here do not correlate with internal metrics of AF3 model confidence such as pLDDT or PAE. While neither of these two metrics match the absolute orientational information encoded in the RDCs, other internal measures of model quality such as crystallographic resolution or crystallographic free R-factor have shown correlations with the fidelity of the RDC fits.^45^

Compared to our AI-based models of RNA with r.m.s. orientational errors of 16° ± 5°, the orientational errors for the DNA AI-based predictions are slightly lower at 14° ± 4°. We also observe, in the case of Drew-Dickerson DNA, possible effects of model training on structural data including crystallization artifacts. The lack of correlation of the orientational error with AF3 confidence metrics (Supporting Information Figure 2), including pLDDT and PAE (Figure 11 and Table 3 Supporting Information), and weakness of its correlation with model precision complicate mistake detection without external data. Overall, AI-based models appear to mimic the training set rather than capture the underlying reality. This departure from ground truth appears consistent with recent observations of collapses of AI models upon recursive training^46^ and is also consistent with recent analysis via X-ray crystallography.^47^ To counter this effect, we recommend including the ability to integrate experimental data in the AI-based predictions, or broadening the set of predicted models to allow postselection against experimental data.

## Data Availability

The data and software underlying this article are available in NIST MIDAS data archiving system at https://github.com/usnistgov/AI-Structure-Prediction. Programs used to protonate some PDBs are available for free as part of AmberTools and accessible at https://ambermd.org/AmberTools.php.

## Supporting Information Available

Supporting Information includes figures of conformational statistics, analysis of helicoidal parameters, and correlation plots of confidence metrics, as well as all nucleic acid sequences used for predictions (PDF)
