# Supplementary material for: Critical Assessment of RNA and DNA Structure Predictions via Artificial Intelligence: The Imitation Game
Source: J Chem Inf Model. 2025 Mar 30;65(7):3544–54. doi: 10.1021/acs.jcim.5c00245 (PMC12004532; doi:10.1021/acs.jcim.5c00245)
Supplement: Supplementary file 1 — ci5c00245_si_001.pdf [file ci5c00245_si_001.pdf]

Supporting Information for

Critical assessment of RNA and DNA structure predictions via artificial intelligence: the imitation game

Christina Bergonzo[1,2] and Alexander Grishaev[1,2]

[1] Biomolecular Measurement Division, Material Measurement Laboratory, National Institute of Standards and Technology, Gaithersburg, Maryland, 20899, USA

[2] Institute for Bioscience and Biotechnology Research, Rockville, Maryland, 20850, USA

Supporting Figure 1. Geometry of Varkud SLV RNA with various added ion buffers.

Supporting Table 1. Conformational statistics for Varkud SLV RNA

Supporting Figure 2. Geometry of the predicted G-quadruplex-hairpin transition.

Supporting Table 2. Extended statistics for the RDC-based AI-predicted model validation for the test set of 28 RNA and DNA constructs.

Supporting Figure 3. Plots of r.m.s. orientational errors correlated with AF3 confidence metric, translational errors, and model precision.

Supporting Figure 4. Comparison of the groove width profiles for the NMR-deposited and AI predicted models for PDB ID: 2KYD.

Supporting Figure 5. RDC validation results for the stem and stem-loop nucleotides of PDB ID: 1NBR.

Supporting Figure 6. RDC validation results for the stem and loop nucleotides of PDB ID: 2M22.

Supporting Figure 7. Comparison of the groove width profiles for the NMR-deposited and AI predicted models of PDB IDs: 2KE6, 2KUR, 2KUU, 2KUV, and 2KUW.

Supporting Figure 8. Comparison of the groove width profiles for the NMR-deposited and AI predicted models of PDB IDs: 5UZD and 5UZF.

Supporting Figure 9. Comparison of the base step tilt, roll, and groove width profiles for the NMR-deposited and AI predicted models of PDB IDs: 1RVH and 1RVI.

Supporting Figure 10. Comparison of the base step tilt, roll, and groove width profiles for the NMR-deposited and AI predicted models of PDB ID: 1NAJ.

Supporting Figure 11. Correlation plots of AI prediction confidence metrics with RDC Q and RMS orientation error

Supporting Table 3. AI Predicted Statistics for the test set of 28 RNA and DNA constructs.

Supporting Text. Nucleotide sequences for all studied systems.

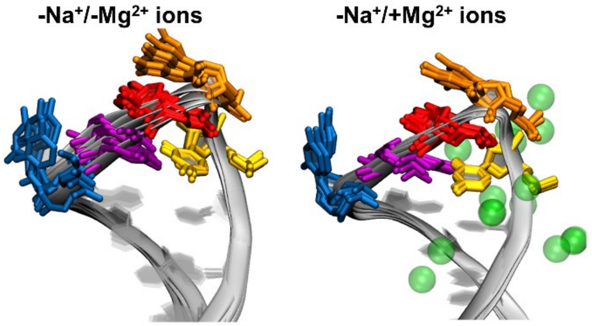

Supporting Figure 1. SLV RNA of Varkud satellite ribozyme determined via AI prediction without ions (left column) and with Mg^{2+} ions (right column), The UNR motif is colored in yellow, orange, and red, respectively, and the rest of the coloring matches a previous publication (Bergonzo et al. 2015). Mg^{2+} ions are colored green.

Supporting Table 1. U-turn characteristics of SLV RNA. Averages and standard deviations are calculated for the NMR (1TBK and 1YN2) and the AI-predicted ensembles. Greyed text corresponds to Main Text Table 1 data.

U-turn Characteristic	AI Predicted (no ions)	1TBK NMR (+Na^+/-Mg^{2+})	AI Predicted (+Na^+/-Mg^{2+})	1YN2 NMR (+Na^+/+Mn^{2+})	AI Predicted (+Na^+/+Mg^{2+})	AI Predicted (-Na^+/+Mg^{2+})
Turn residue N α (degree)	157 ± 3	116 ± 6	194 ± 47	184 ± 38	168 ± 2	291 ± 2
Stacking bases after turn (Å) N, R	3.93 ± 0.32	3.63 ± 0.19	3.56 ± 0.05	4.22 ± 0.44	3.63 ± 0.03	4.45 ± 0.12
Stacking bases after turn (Å) R, R + 1	4.36 ± 0.26	3.65 ± 0.30	3.78 ± 0.06	3.80 ± 0.24	3.86 ± 0.07	4.54 ± 0.09
Stacking U base and R 5′-phosphate (Å)	4.26 ± 0.21	4.16 ± 0.36	4.48 ± 0.27	3.90 ± 0.64	4.66 ± 0.23	10.24 ± 0.07
H-bond between U 2′OH and R N7 (Å)	2.81 ± 0.21	3.36 ± 0.29	3.27 ± 0.16	2.55 ± 0.17	2.95 ± 0.07	8.63 ± 0.16
U N3 and R 3′-phosphate distance (Å)	5.41 ± 0.27	8.78 ± 0.31	4.18 ± 0.15	5.14 ± 0.68	4.33 ± 0.14	13.37 ±0.08

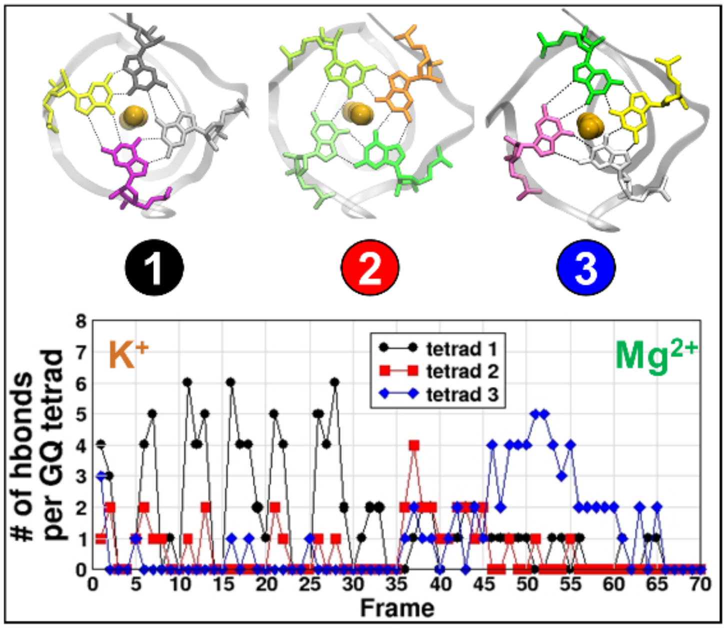

Supporting Figure 2. Top: three tetrads formed in AI-based prediction of G-quadruplex. Bottom: Plot showing the number of hydrogen bonds per G-quadruplex tetrad (tetrads 1, 2, and 3 are colored black, red, and blue and match label on top of this figure) for frames with increasing numbers of Mg^{2+} ions and decreasing numbers of K^{+} ions. No tetrad ever adopts all eight expected hydrogen bonds.

Supporting Table 2. Extended statistics for the RDC-based AF3 model validation for the test set of 28 RNA and DNA constructs.

PDB ID	Total nts.	N_{RDC}	Type	Loop	r.m.s. orient. error via Q (°)	r.m.s. orient. error via rms (°)
**RNA:**						
2kyd	32	42	stem	none	7.0	6.1
$2gbh_{stem}$	16	78	stem	none	7.8	8.1
2k4c	76	24	4 helices	multiple	10.1	10.0
2koc	14	30	stemloop	UUCG	10.3	9.9
2k5z	29	98	stemloop	UGUU	11.1	10.2
1p5n	34	105	stemloop	AGCC	13.3	12.7
2m21	21	47	stemloop	CACUAUU	14.2	12.7
2rn1	32	89	stemloop/stemloop	CUGGGA/UCCCAG	14.0	12.5
1nbr	29	127	stemloop	CAGUGC	13.4	15.3
2m22	23	62	stemloop	GUAAU	15.2	14.0
2kuw	48	103	stemloop	AUUAAUUC	15.7	14.7
1xhp	32	76	stemloop	GCAUA	15.8	16.7
2kuu	48	117	stemloop	AUUAAUUC	16.9	16.2
2kuv	48	95	stemloop	AUUAAUUC	17.9	17.5
2m8k	48	91	pseudoknot		17.9	18.5
5a17	32	75	stemloop	AUCAA	17.7	19.3
1z31	32	65	stem-loop-stemloop	UUCG	18.8	18.7
2kur	48	100	stemloop	AUUAAUUC	19.4	18.7
1p5m	55	136	stem-loop-stemloop	UUCG	20.8	19.5
2ke6	48	115	stemloop	AUUAAUUC	20.6	19.8
$2gbh_{loop}$	8	44	loop	GΨUGAAAA	22.0	22.8
1jox	21	54	stemloop	UGAGAU	30.2	28.8
**DNA:**						
5uzf	24	67	B-DNA stem	none	9.8	9.1
5uzd	24	57	B-DNA stem	none	10.4	9.1
1rvh	24	72	B-DNA stem	none	11.9	11.2
1fzx	24	84	B-DNA stem	none	13.6	12.8
1g14	24	79	B-DNA stem	none	15.9	14.4
1rvi	24	68	B-DNA stem	none	18.8	17.4
1naj	24	60	B-DNA stem	none	18.7	18.3

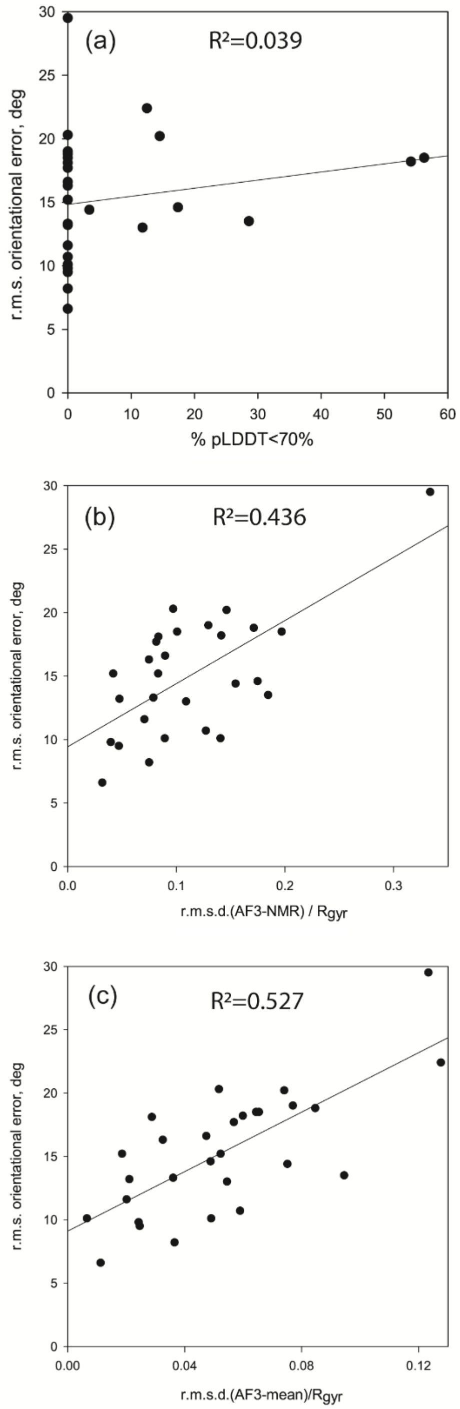

Supporting Figure 3. Correlations of the AI-predicted models' r.m.s. orientational errors with (a) the percentages of nucleotides predicted with low confidence, (b) the ratios of the coordinate r.m.s.d. between the AI-predicted and NMR-deposited models and the gyration radius, and (c) the ratios of the AI-predicted models' r.m.s. to the mean and the gyration radius. Correlation coefficients are reported as R^2.

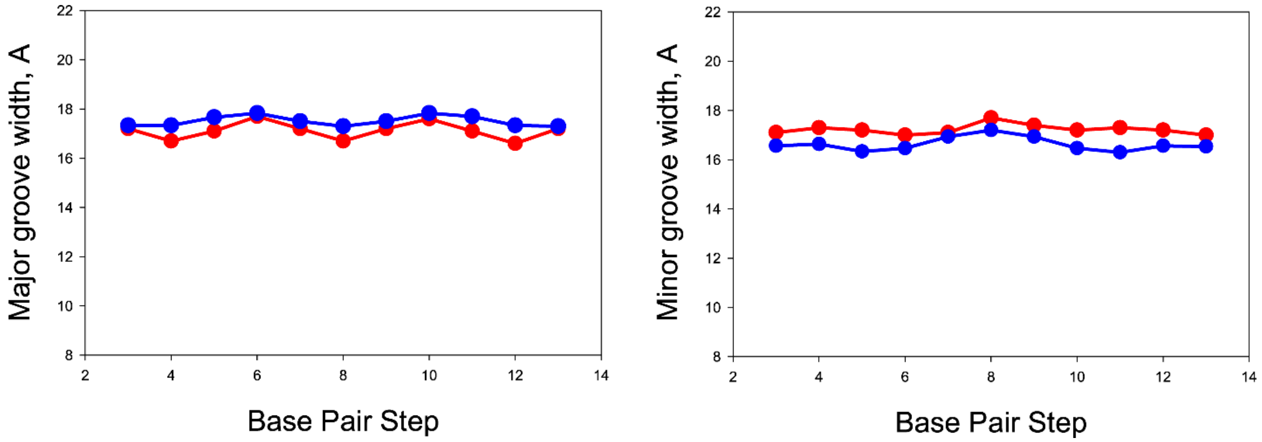

Supporting Figure 4. Groove width profiles via P-P distances for the representative model of the PDB ID: 2KYD (red) and the respective AI-based predictions (blue). The averages for the first AF3 models are shown. Calculations were performed with 3DNA software (http://web.x3dna.org/).

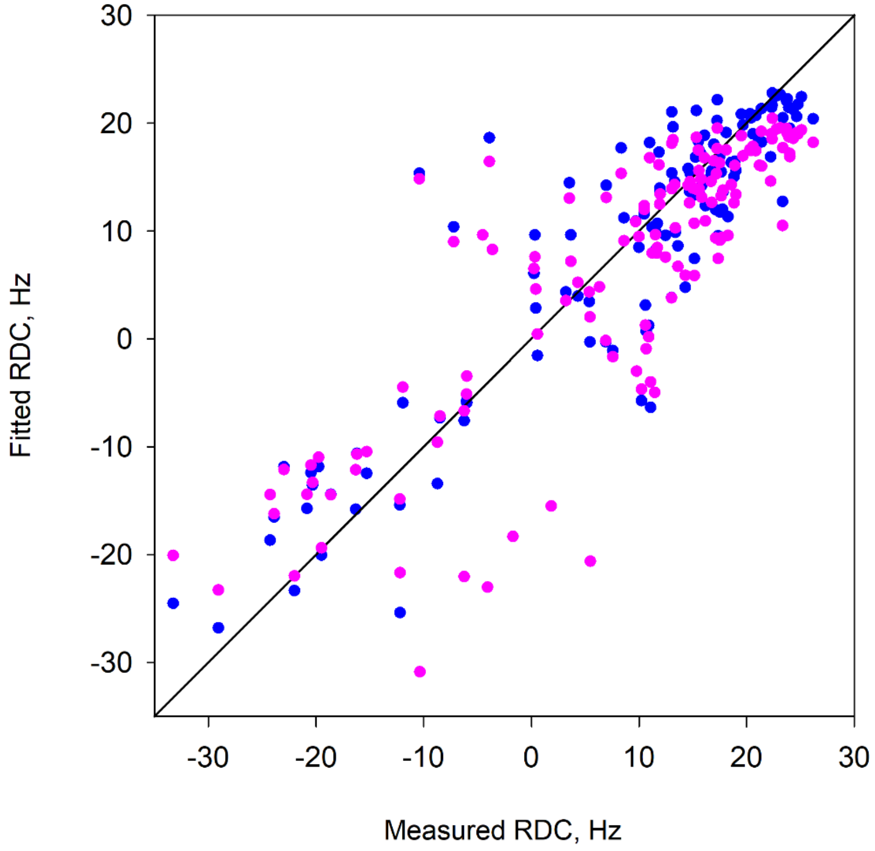

Supporting Figure 5. The agreement of the AI-predicted models for PDB ID: 1NBR with the experimental RDCs for the stem (blue, Q=0.318, nts. 1-9 and 15-23) and the entire stem-loop (pink, Q=0.453, nts. 1-23).

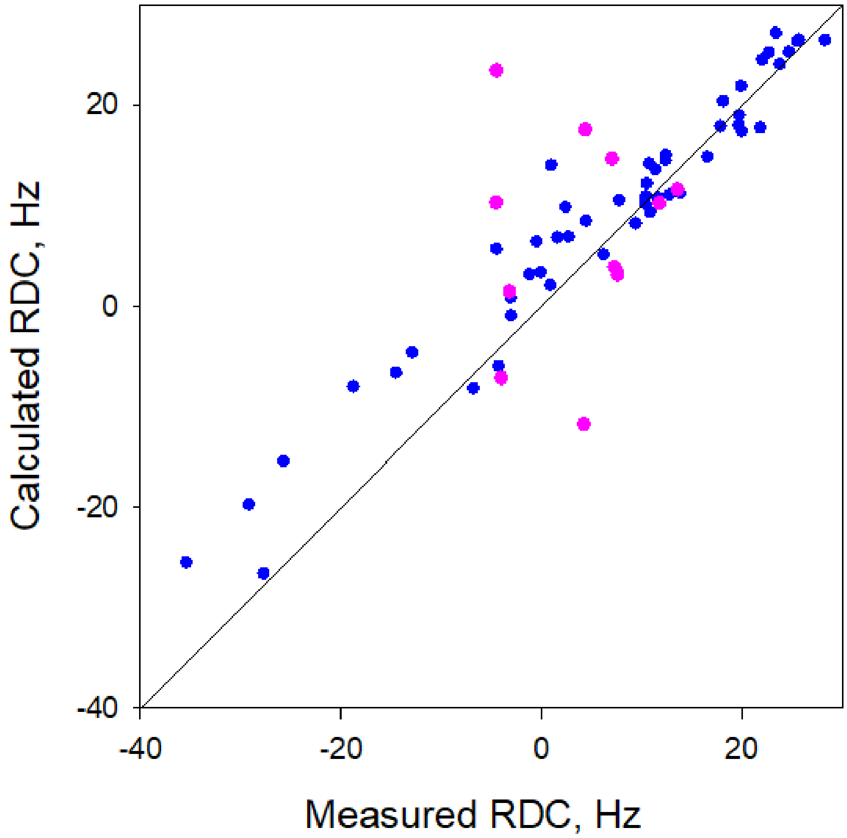

Supporting Figure 6. The agreement of the AI-based models for PDB ID: 2M22 with experimental RDCs. Fitted stem RDCs are shown in blue (Q=0.271) and the predicted loop RDCs in pink (Q=0.671).

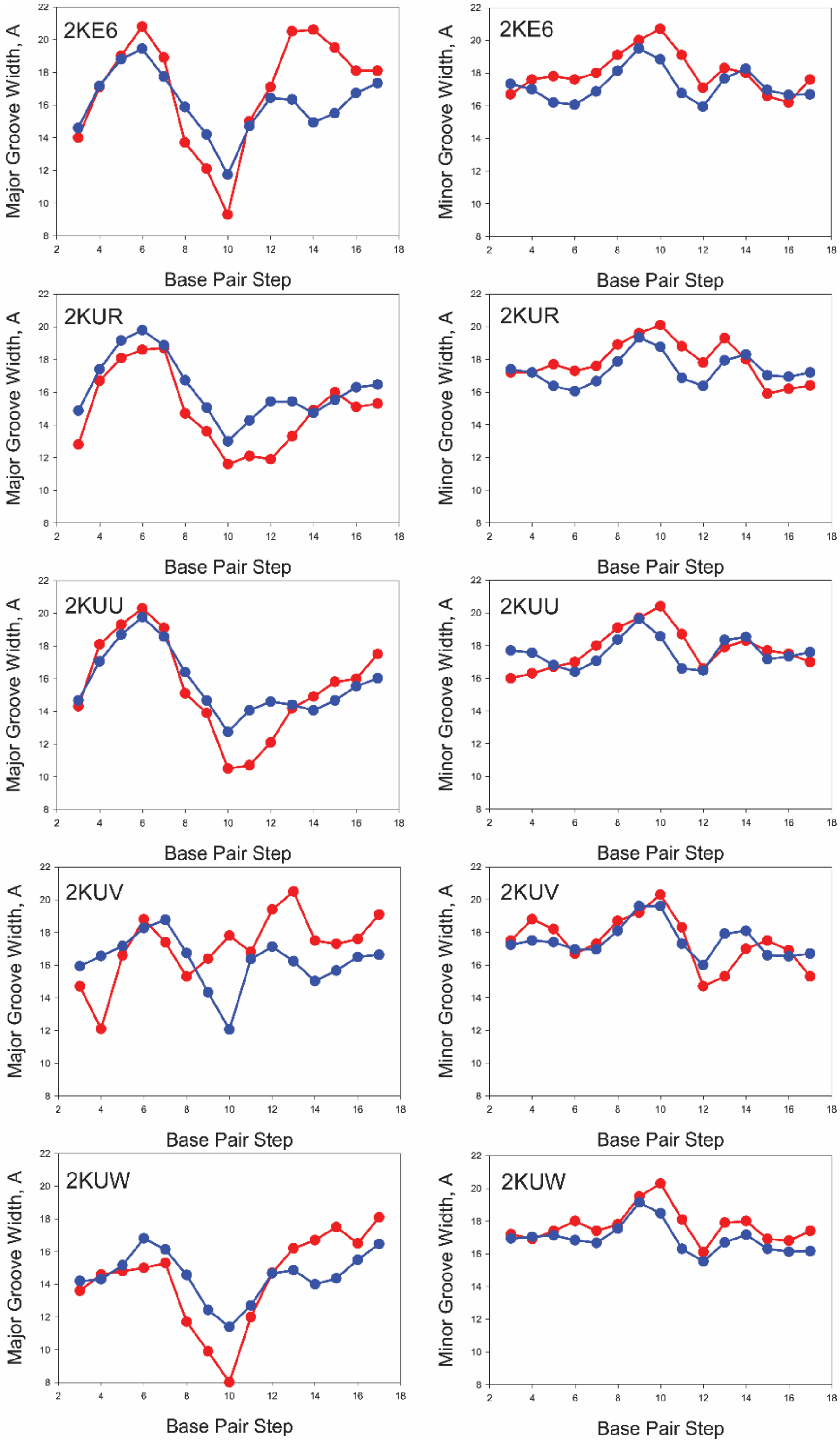

Supporting Figure 7. Groove width profiles via P-P distances for the representative models of the NMR structures (red) and the respective predictions (blue) for PDB IDs: 2KE6, 2KUR, 2KUU, 2KUV, 2KUW. The averages for the first three models are shown. Calculations were performed with 3DNA software (http://web.x3dna.org/).

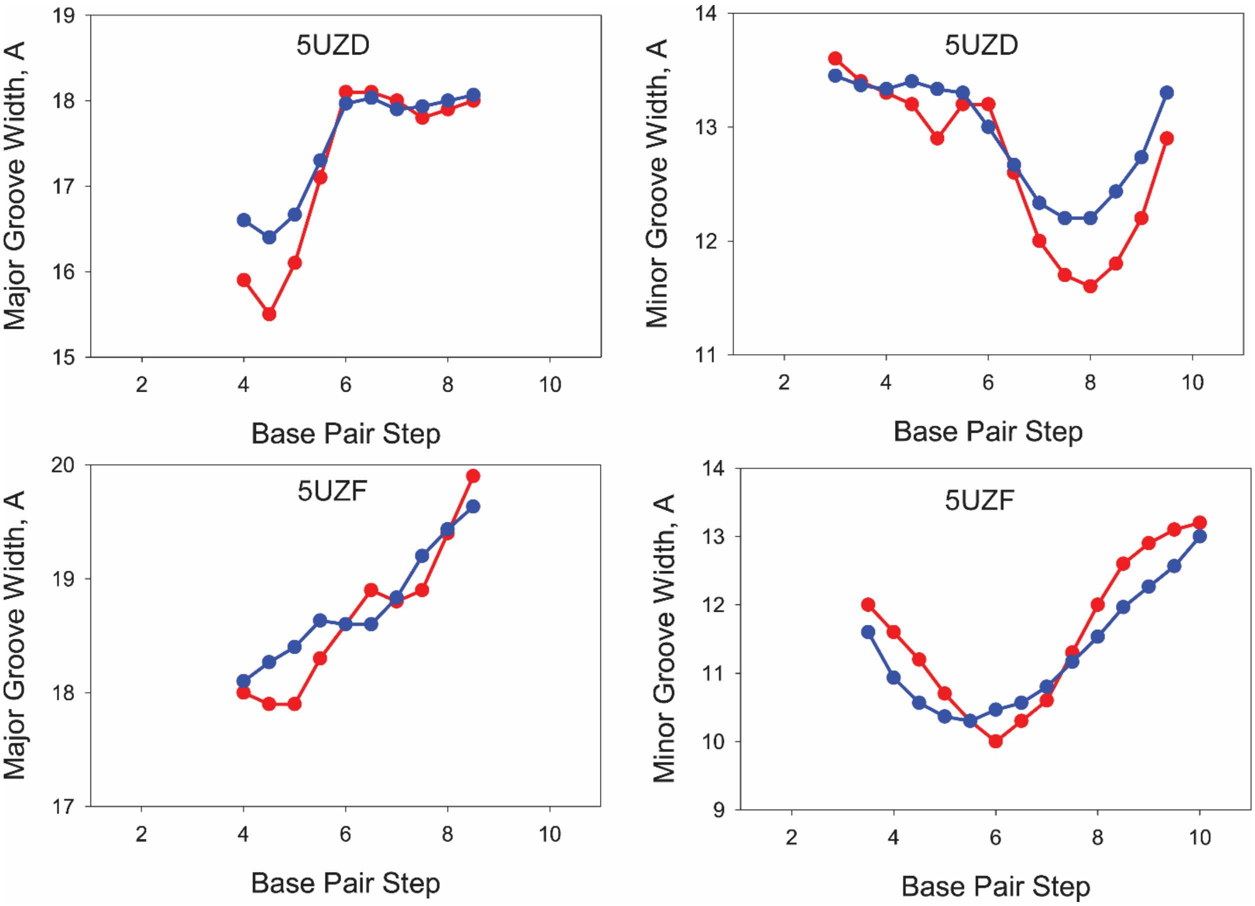

Supporting Figure 8. Groove width profiles via P-P distances for the representative models of the NMR structures (red) and the respective AI-based predictions (blue) for PDB IDs: 5UZD and 5UZF. The averages for the first three models are shown. Calculations were performed with the Curves+ software, with 5.8 Å added to the software-reported groove width parameters.

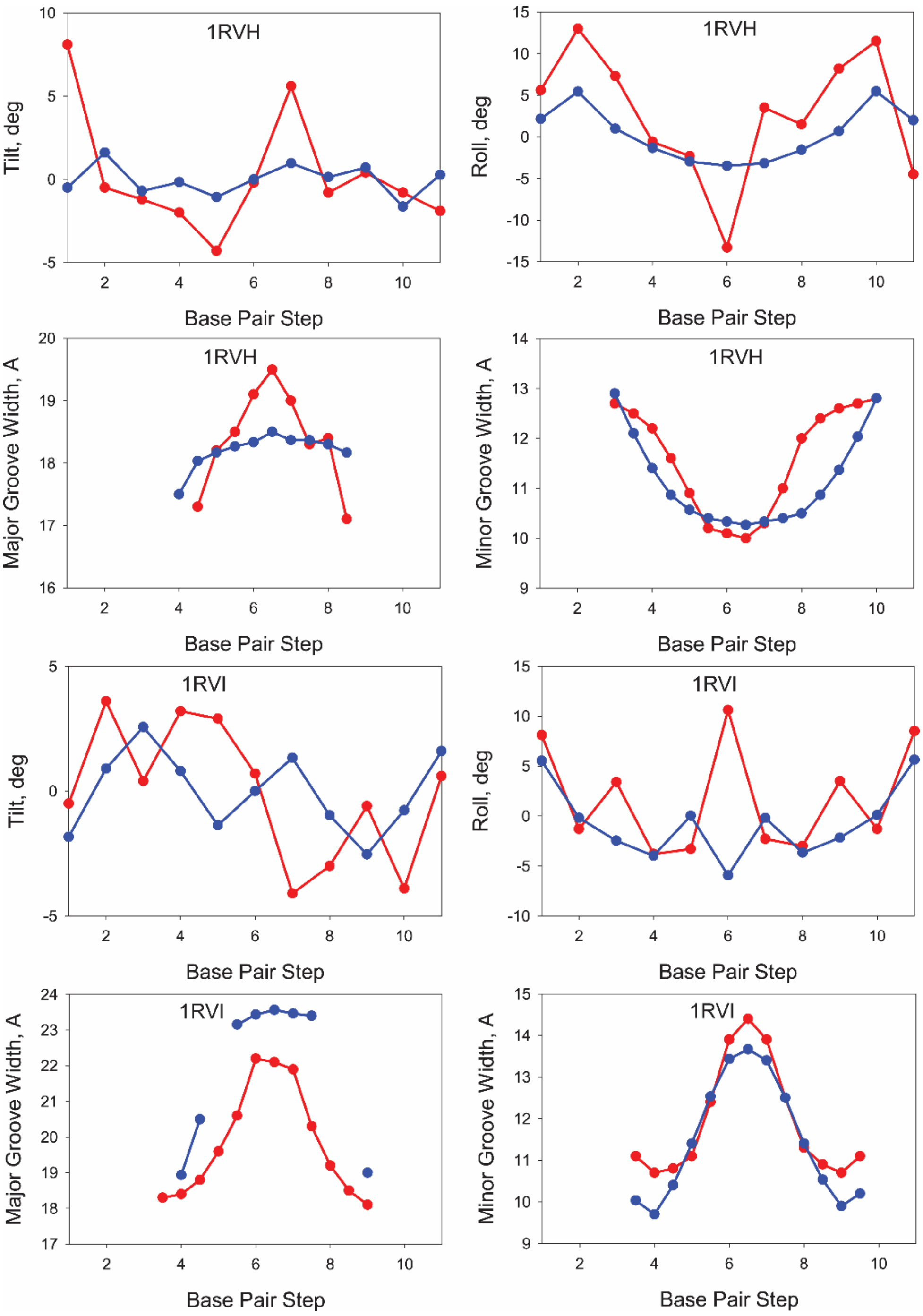

Supporting Figure 9. Comparison of the tilt, roll, and groove widths profiles for the most representative models of the 1RVH and 1RVI depositions (red) and the corresponding predictions (blue). The averages for the first three models are shown. Calculations were performed with the Curves+ software, with 5.8 Å added to the software-reported groove width parameters. Missing data points are due to Curves+ not reporting the corresponding values.

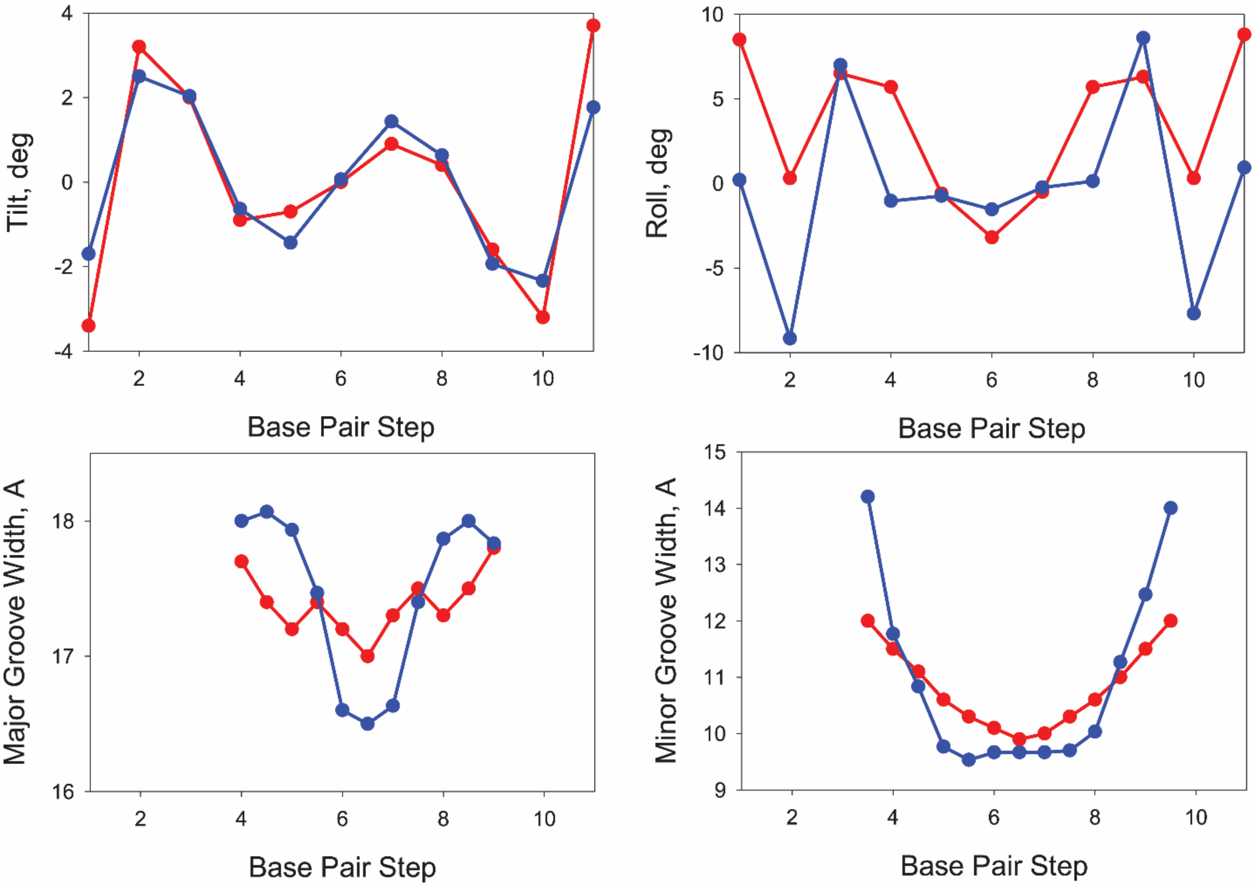

Supporting Figure 10. Comparison of the tilt, roll, and groove widths profiles for the most representative model of the 1NAJ deposition (red) and the corresponding predictions (blue). The averages for the first three models are shown. Calculations were performed with the Curves+ software, with 5.8 Å added to the software-reported groove width parameters.

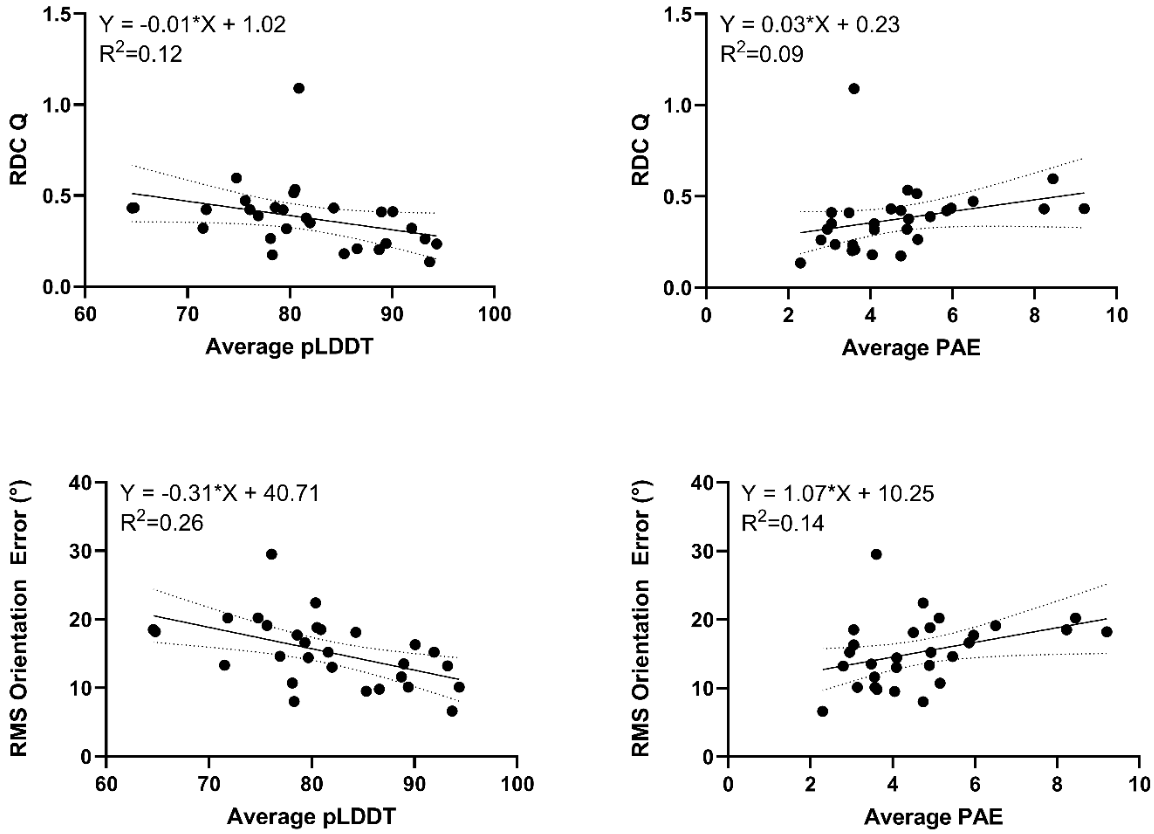

Supporting Figure 11. Correlation plots of AI prediction confidence metrics pLDDT (per-atom confidence estimate, left columns) and PAE (predicted aligned error, right columns) versus RDC Qfree (top row) and RMS orientation error (bottom row) for 28 systems. The equation of a linear fit is shown at the top left-hand corner, with R^2 goodness of fit. Linear fits and 95% confidence intervals are shown on each plot.

Supporting Table 3. AI Predicted Statistics for the test set of 28 RNA and DNA constructs.

**RNA:**				
	Average per residue pLDDT (%)	StDev	Average per residue PAE (Å)	StDev
2kyd	93.68	1.96	2.30	1.05
2k4c	94.36	3.87	3.57	1.99
2koc	89.41	4.14	3.14	1.69
2k5z	78.12	4.91	5.15	2.86
1p5n	81.99	6.94	4.09	2.51
2m21	71.53	9.17	4.89	2.89
2rn1	88.96	2.66	3.48	1.75
1nbr	79.69	5.65	4.10	2.12
2m22	76.92	4.87	5.46	2.88
2kuw	81.61	3.93	4.93	2.85
1xhp	90.09	2.78	3.05	1.58
2kuu	79.35	4.51	5.86	3.45
2kuv	78.58	5.15	5.97	3.69
2m8k	64.80	14.94	9.21	5.28
5a17	64.60	10.60	8.23	4.41
1z31	80.53	4.80	4.91	2.46
2kur	75.66	5.84	6.50	4.13
1p5m	74.78	9.83	8.45	5.03
2ke6	80.39	5.02	5.13	2.99
1jox	80.89	6.12	3.60	2.25
**DNA:**				
	Average per residue pLDDT (%)	StDev	Average per residue PAE (Å)	StDev
5uzf	85.32	4.78	4.05	2.40
5uzd	86.58	3.96	3.62	1.85
1rvh	88.73	4.15	3.56	2.06
1fzx	93.24	4.08	2.80	1.65
1g14	91.91	4.00	2.95	1.74
1rvi	84.29	4.57	4.50	2.73
1naj	93.24	4.27	3.05	1.91

Nucleotide sequences of 28 RNA and DNA constructs used for RDC-based validation

2KYD:
CUAGUUAGCUAACUAG/CUAGUUAGCUAACUAG

2GBH:
GGGCUAAUGYUGAAAAAUUAGCCC

2K4C:
GGGUGAUUAGCUCAGCUGGGAGAGCACCUCCCUUACAAGGAGGGGGUCGGCGG
UUCGAUCCCGUCAUCACCCACCA

2K5Z:
GGUCUACAUUGCUGUUGUCGUGUGUGACC

2KOC:
GGCACUUCGGUGCC

1P5N:
GGCAGAAAGCGUCUAGCCAUGGCGUUAGUAUGCC

2M21:
GGCGAUACACUAUUUAUCGCC

2RN1:
GAGCCCUGGGAGGCUC/GCUGGUCCCAGACAGC

1NBR:
GGAGUGCUUCAACAGUGCUUGGACGCUCC

2M22:
GGCAGAUCUGUAAUAGAACUGCC

2KUW:
GGGGUUGGUGUAUUUUUAAAUUAAUUCUUAAAAACUACAAUCAGCUCC

1XHP:
GAGCAGUUCCCCUGCAUAAGGAUGAACCGUUC

2KUU:
GGCUUGAUUGUAUGUGUAAAUUAAUUCUUACACACUACAAAUUAAGCC

2KUV:
GGCUCGAUUGUAUUUUUAAAUUAAUUCUUAAAAACUACAAAUCGAGCC

2M8K:
GGUUUCUUUUUAGUGAUUUUUCCAAACCCCUUUGUGCAAAAAUCAUUA

5A17:
GACGAUAUCGAGCAUCAAGAGUGAAUAUCGUC

1Z31:
GAGGUCGGCCCGACUUCGGUCACUGCCACCUC

2KUR:
GGCUUGAUUGUAUUAUUAAAUUAAUUCUUAAUAACUACAAAUUAAGCC

1P5M:
GGCUGUGAGGAACUACUGUCUUCACGCCUUCGGGAGUGUCGUGCAGCCUCCAG
CC

2KE6:
GGCUUGAUUGUAUUUUUAAAUUAAUUCUUAAAAACUACAAAUUAAGCC

1JOX:
GGCGGUGCUGAGAUGCCCGUC

5UZF:
CGATTTTTTGGC/GCCAAAAAATCG

5UZD:
GCATCGATTGGC/GCCAATCGATGC

1RVH:
GCAAAATTTTGC/GCAAAATTTTGC

1FZX:
GGCAAAAAACGG/CCGTTTTTTGCC

1G14:
GGCAAGAAACGG/CCGTTTCTTGCC

1RVI:
CGTTTTAAAACG/CGTTTTAAAACG

1NAJ:
CGCGAATTCGCG/CGCGAATTCGCG

Nucleotide sequences of 7 RNA constructs used for evaluations of ion dependence and termini sequence dependence

1TBK/1YN2:
GCGAGUUGACUACUCGC

Hairpin/G-quadruplex:
AGGGAGGGGCGGGAGUGGGCUACCCGC

PreQ1:
AGAGGUUCUAGCACAUCCCUCUAUAAAAAACUAA

MAPT 10 exon 25mer:
GGCAGUGUGAGUACCUUCACACGUC

MAPT 10 exon 30mer:
GGCGGCAGUGUGAGUACCUUCACACGUCCC

Cap1G-TPUD:
GGUCUCUCUUGUUAGACCAGGUCGAGCCCGGGAGCUCUCUGGCUAGCAAGGAA
CCCACUGCUUAAGCCUCAAUAAAGCUUGCCUUGAGUGCCUCAAGCAGUGUGUG
CCCGAGAGAGGUGCACACAGCAAGAGGCGAGAGC

Cap3G-TPUD:
GGGGUCUCUCUUGUUAGACCAGGUCGAGCCCGGGAGCUCUCUGGCUAGCAAGG
AACCCACUGCUUAAGCCUCAAUAAAGCUUGCCUUGAGUGCCUCAAGCAGUGUGU
GCCCGAGAGAGGUGCACACAGCAAGAGGCGAGAGC
